# Doxorubicin-Loaded Polymeric Micelles Conjugated with CKR- and EVQ-FLT3 Peptides for Cytotoxicity in Leukemic Stem Cells

**DOI:** 10.3390/pharmaceutics14102115

**Published:** 2022-10-04

**Authors:** Fah Chueahongthong, Singkome Tima, Sawitree Chiampanichayakul, Pornngarm Dejkriengkraikul, Siriporn Okonogi, Mathurada Sasarom, Soraya Rodwattanagul, Cory Berkland, Songyot Anuchapreeda

**Affiliations:** 1 Department of Medical Technology, Faculty of Associated Medical Sciences, Chiang Mai University, Chiang Mai 50200, Thailand; 2 Research Center of Pharmaceutical Nanotechnology, Chiang Mai University, Chiang Mai 50200, Thailand; 3 Cancer Research Unit of Associated Medical Sciences (AMS-CRU), Chiang Mai University, Chiang Mai 50200, Thailand; 4 Department of Biochemistry, Faculty of Medicine, Chiang Mai University, Chiang Mai 50200, Thailand; 5 Department of Pharmaceutical Sciences, Faculty of Pharmacy, Chiang Mai University, Chiang Mai 50200, Thailand; 6 Department of Pharmaceutical Chemistry, School of Pharmacy, University of Kansas, Lawrence, KS 66047, USA

**Keywords:** doxorubicin, CKR, EVQ, polymeric micelle, leukemic stem cell, co-treatment

## Abstract

Doxorubicin (Dox) is the standard chemotherapeutic agent for acute myeloblastic leukemia (AML) treatment. However, 40% of Dox-treated AML cases relapsed due to the presence of leukemic stem cells (LSCs). Thus, poloxamer 407 and CKR- and EVQ-FLT3 peptides were used to formulate Dox-micelles (DMs) and DM conjugated with peptides (CKR and EVQ) for improving AML-LSC treatment. Results indicated that DMs with a weight ratio of Dox to P407 of 1:200 had a particle size of 23.3 ± 1.3 nm with a high percentage of Dox entrapment. They were able to prolong drug release and maintain physicochemical stability. Following effective DM preparation, P407 was modified and conjugated with FLT3 peptides, CKR and EVQ to formulate DM-CKR, DM-EVQ, and DM-CKR+DM-EVQ. Freshly synthesized DMs displaying FLT3 peptides showed particle sizes smaller than 50 nm and a high drug entrapment level, comparable with DMs. DM-CKR+DM-EVQ was considerably more toxic to KG-1a (AML LSC-like cell model) than Dox-HCl. These FLT3-targeted DMs could increase drug uptake and induce apoptosis induction. Due to an increase in micelle-LSC binding and uptake, DMs displaying both peptides tended to improve the potency of Dox compared to a single peptide-coupled micelle.

## 1. Introduction

Feline McDonough Sarcoma (FMS)-like tyrosine kinase or FLT3 protein is a member of the class III receptor tyrosine kinase (RTK) family [1,2]. These RTKs share a common structure consisting of five extracellular immunoglobulin-like domains, a single transmembrane (TM) domain, a cytoplasmic juxtamembrane (JM) region, and two cytoplasmic tyrosine kinase domains (TKD; TK1 and TK2) interrupted by the kinase insert. FLT3 is expressed in a variety of human cells and organs, such as hematopoietic cells, placenta, gonads, and brain. In normal bone marrow, expression appears to be restricted to early progenitors, including CD34^+^ cells. It is primarily expressed on committed myeloid and B-lymphoid progenitors and plays a key role in their survival, proliferation, and differentiation [3,4]. Low levels of FLT3 protein expression have been found in normal peripheral blood mononuclear cells (PBMCs). In contrast, the overexpression of FLT3 protein has been found in leukemic blood cells, especially in AML (acute myeloblastic leukemia) and B-cell acute lymphoid leukemia (B cell ALL) [5]. Previous studies reported that FLT3 and its ligand (FLT3 ligand; FL) play a crucial role in the survival or proliferation of leukemic blast cells [6,7]. In addition, FLT3 mutations, including internal tandem duplication in JM domain (FLT3-ITD) and a missense point mutation at the D835 residue within a FLT3 tyrosine kinase domain (FLT3-TKD), have been associated with a poor prognosis for overall survival [8]. Both FLT3 mutations and FLT3 expression levels have been used as biological markers for diagnosis and evaluation of the prognosis of leukemia. All these findings supported that FLT3 gene expression may influence leukemogenesis, especially in AML.

One theory of relapse in AML patients involves the presence of subpopulations of leukemic stem cells (LSCs) [9]. The LSCs have been defined as human AML initiating cells with self-renewal capacity and the ability to give rise to heterogeneous lineages of cancer cells [10,11,12]. LSCs can be identified by the cell surface phenotype CD34^+^ hematopoietic stem cell and CD38^−^ subpopulation [13]. The use of traditional drugs is quite ineffective on the LSC population due to three major causes. First, chemotherapeutics have typically been designed to eliminate fast-dividing cells by inhibiting cell cycle progression leading to the arrest of cancer cell multiplication [14]; they do not affect LSCs that reside mostly in a quiescent state of cell cycle [15]. Second, LSCs express multidrug-resistant efflux pump proteins, such as P-glycoprotein (P-gp), that can remove potentially cytotoxic chemotherapeutic agents from the cell [16,17]. Third, LSCs may undergo mutations and epigenetic changes that can reduce toxicity and lead to resistance to conventional drugs [18,19]. Thus, LSCs are considered to play a fundamental role in AML pathogenesis and have become a main target for therapy of AML.

Doxorubicin (14-hydroxydaunorubicin; C_27_H_29_NO_11_) or Dox is a second-generation anthracycline chemotherapeutic drug that has been widely used for over 40 years to treat various hematological and solid tumor malignancies [20]. The reported anticancer activity involves interaction with nuclear components. Dox can intercalate into DNA and bind to non-histone proteins such as type II topoisomerases, causing double strand DNA breaks, and histone proteins such as histone linker, resulting in chromatin unfolding and aggregation. Additionally, it generates oxidative stress and strong DNA damaging OH-radicals, leading to apoptosis [21]. However, due to the limitations of Dox in treatment-resistant malignancies, especially cancer stem cells, and systemic toxicity, a drug delivery system such as liposomes has been explored to alleviate these adverse effects [20,21,22]. Dox is the standard chemotherapy for AML, which is highly effective at eradicating leukemic blast cells. However, 40% of Dox-treated AML cases relapsed due to drug-resistant proteins and the existence of LSCs which are generally quiescent in the G_0_-stage of the cell cycle. In this study, Poloxamer 407 (P407), a triblock co-polymer, was selected to formulate micelles. Additionally, to improve Dox transport to LSCs and improve its safety at relatively high drug dosages, P407 was further modified and conjugated with FLT3-specific peptides (CKR and EVQ) which were designed by Tima et al. (2016) [23].

Over the years, drug delivery systems, including liposomes, micelles, hydrogel, and nanoparticulate systems have been proposed to overcome the adverse effects of Dox in many cancers, such as breast cancer, lung cancer, melanoma, and glioblastoma [24,25,26,27]. Polymeric micelles are often tested as nanoscale core structures for solubilization, stabilization, and insoluble drug delivery in pharmaceutical applications [28]. Several studies have reported the application of polymeric-micelle based Dox against cancer and CSC, e.g., polymeric micelle formulation of Dox with a Pluronic L61 and F127 mixture, SP1049C for the treatment of CSC in breast cancers [29], co-delivery of pyrrolidinedithiocarbamate (PDTC) and Dox loaded in copolymer folate-chitosan for overcoming multidrug-resistant liver cancer cells [30], and Dox-loaded HA-g-PLGA micelles for the treatment of human colorectal carcinoma [31]. P407, also known as Pluronic F127, is a copolymer with a molecular weight of approximately 12.6 kDa (PEO101-PPO56-PEO101) with a polyoxyethylene content of 70%, which contributes to its hydrophilicity [32]. It is a non-ionic surfactant with a high solubilizing capacity, low toxicity, good drug release properties, and compatibility with cells, body fluids, and a wide range of chemicals [32]. Moreover, it can directly enter cells and modulate numerous biological processes [33]. P407 and other poloxamers such as P188 were employed as a material to develop Dox-loaded nanocarriers such as hydrogel, liposome, or co-treatment with Dox for lung cancer and hepatocarcinoma therapies [34,35,36]. Due to the features of P407, it was selected to prepare the core-shell structure of polymeric micelles in this research.

To enhance the effectiveness of Dox-loaded micelles against FLT3-positive AML cell lines, FLT3-specific peptides were designed based on the amino acid sequence of FLT3 ligand (FL) and the binding sites of the FLT3 receptor and its ligand (FL). CKR1 and EVQ sequences are CKRFQNSHL (C_48_H_77_N_17_O_13_S_1_) and EVQTCISHLL (C_49_H_83_N_13_O_16_S_1_), respectively. According to the docking results obtained from our previous study [23,37], the binding sites of CKR1 and EVQ peptides are related to domains 2 and 3 of the FLT3 extracellular domain. The binding of both peptides to an extracellular domain of the FLT3 protein receptor around the high-affinity binding site and the high number of hydrogen bonds used at the binding site makes both FLT3-specific peptides attractive for targeting the FLT3 protein on the leukemic cell membrane.

In this study, Dox-loaded polymeric micelles were developed and conjugated with the CKR1 (CKR) and EVQ peptides, which were selected as representatives FLT3 peptides, and assessed for physiochemical properties. After that, Dox-micelles (DMs) displaying both FLT3 CKR- and EVQ-conjugated peptides or the individual peptides were compared to Dox aqueous solution by testing for cytotoxic effects and cell apoptosis induction in FLT3 expressing leukemic stem cells (KG-1a) [38] and FLT3 expressing leukemic cells (EoL-1). The CKR- and EVQ-conjugated Dox-loaded polymeric micelles displaying both peptides were hypothesized to increase cytotoxicity in leukemic stem cells.

## 2. Materials and Methods

### 2.1. Materials

Doxorubicin hydrochloride injection IP 50 mg/25 mL (ADRIM, Fresenius Kabi, BKK, Thailand) was purchased from Maharaj Nakhon Chiang Mai Hospital. Doxorubicin hydrochloride salt was purchased from LC Laboratories (Woburn, MA, USA). Poloxamer 407 (Kolliphor^®^ P407), dichloromethane (DCM; CH_2_Cl_2_) anhydrous, ≥99.8%, Dess–Martin periodinane, deuterated chloroform (CDCl_3_), tetrahydrofuran (THF; C_4_H_8_O) anhydrous, ≥99.9%, inhibitor-free, dimethyl sulfoxide (DMSO; C_2_H_6_OS), trifluoroacetic acid (TFA; C_2_HF_3_O_2_), (+)-sodium L-ascorbate (C_6_H_7_NaO_6_), crystalline, ≥98%, and aminoguanidine hydrochloride (NH_2_NHC(=NH)NH_2_·HCl), ≥98%, were purchased from Sigma-Aldrich (St Louis, MO, USA). Azido-PEG6-amine was purchased from BroadPharm (San Diego, CA, USA). FLT3-specific peptides with N-terminal propargylglycine modification, CKR1 (CKRFQNSHL (C_48_H_77_N_1_7O_13_S_1_), MW 1132.29 g/mol) and EVQ (EVQTCISHLL (C_49_H_83_N_13_O_16_S_1_), MW 1237.00 g/mol), were synthesized by Biomatik company (Wilmington, DE, USA). Ligand THPTA (tris-hydroxypropyltriazolylmethylamine) was purchased from Click Chemistry Tools (Scottsdale, AZ, USA). Sodium triacetoxyborohydride (STAB, C_6_H_10_BNaO_6_), 97%, and petroleum ether 40–60 °C, certified AR for analysis was purchased from Fisher Scientific (Pittsburgh, PA, USA). RPMI-1640 Medium, IMDM (Iscove’s Modified Dulbecco’s Medium), L-glutamine, penicillin/streptomycin, dialysis membrane tubing 3500 Dalton MWCO, 4′,6-diamidino-2-phenylindole (DAPI), and phosphate buffered saline (PBS) pH 7.4, 10× solution was purchased from Thermo Fisher Scientific (Waltham, MA, USA). 3-(4,5-Dimethylthiazol-2-yl)-2,5-Diphenyltetrazolium Bromide (MTT) reagent was obtained from BioVision (Milpitas, CA, USA). Amicon^®^ Ultra 15 mL Centrifugal Filters 4500 Dalton MWCO, Immobilon^®^-P PVDF Membrane, rabbit polyclonal anti-FLT3, rabbit polyclonal anti-GAPDH, and Luminata™ Forte Western HRP Substrate were purchased from MilliporeSigma (Burlington, MA, USA). HRP-conjugated goat anti-rabbit IgG was purchased from Promega (Madison, WI, USA). An FITC Annexin V apoptosis detection kit with PI was obtained from BioLegend (San Diego, CA, USA).

### 2.2. Cell Lines and Cell Culture Conditions

KG-1a (human acute myeloblastic leukemic cell line; ATCC^®^ CCL-246.1™) was purchased from ATCC (Manassas, VA, USA). EoL-1 (human eosinophilic leukemia cell line; RBRC-RCB0641) was purchased from RIKEN BRC Cell Bank (Ibaraki, Japan). Both cell lines were used as myeloid cell line models in this study. KG-1a (model of FLT3-positive stem cell like-leukemic cells) was cultured in IMDM supplemented with 20% fetal bovine serum, 100 units/mL penicillin, and 100 µg/mL streptomycin. EoL-1 (a model of FLT3-positive leukemic cells) was cultured in RPMI-1640 medium containing 10% fetal calf serum, 1 mM L-glutamine, 100 units/mL penicillin, and 100 µg/mL streptomycin. The cells were maintained at 37 °C in a humidified incubator with 5% CO_2_.

### 2.3. Preparation of DMs

The amount of Dox-HCl was set at 0.5 mg while the amount of P407 was varied in many conditions to optimize the most suitable formulation for Dox-micelle synthesis. To produce Dox encapsulated polymeric micelles, a film-hydration technique in cooperation with a pH-induced self-assembly method was performed [39]. In brief, poloxamer P407 was dissolved in methanol for a final concentration of 20 mg/mL. The blank micelle-films were formed by evaporation under reduced pressure in a rotary evaporator at 45 °C, 50 rpm, for 20 min, and then dried at room temperature (RT) overnight. To form DMs, the blank micelle-film was resuspended in normal saline solution (NSS) and stirred at 350 rpm for 30 min at RT. Afterwards, 10×PBS at pH 7.4 and doxorubicin aqueous solution (Dox-HCl) were dropped into the micelle solution at the rate of 200 µL/min while stirring. The final volume ratio for NSS:10×PBS:Dox-HCl was 7:1:2. Following overnight stirring, the DM solution was centrifuged at 12,000 rpm for 10 min, and then filtered through a 0.22 mm filter to obtain DMs. All samples were stored in light-protecting containers.

### 2.4. Investigation of Physicochemical Properties of DMs

The physical properties of DMs, including particle size, polydispersity index (PdI), and zeta potential (ZP), were assessed using NanoBrook 90-Plus Zeta dynamic light scattering nanoparticle size analyzer (Brookhaven Instruments Corporation, Holtsville, NY, USA) and Zetasizer Nano ZS (Malvern Panalytical, Malvern, UK). The morphology of DMs was determined using a Hitachi H-8100 Transmission Electron Microscope (Hitachi High-Technologies Corporation, Tokyo, Japan). For evaluating Dox content in DMs, the samples were mixed at a volume ratio of 1:1 with methanol and centrifuged at 5000 rpm for 5 min to dissociate the micelles. The Dox concentration was then quantified using a reverse-phase HPLC (RP-HPLC) (Waters Alliance HPLC system equipped with a dual-wavelength ultraviolet-visible (UV-vis) detector, Waters Corporation, Milford, MA, USA) with linear gradient chromatographic conditions of 5–95% acetonitrile in water (with constant 0.05% trifluoroacetic acid) over 20 min, with a Waters XBridge column (C18, 5 μm, 4.6 × 250 mm), a flow rate of 1.0 mL/min and detection at 280 nm, or using a UV-visible spectrophotometer at 480 nm (Agilent 8453 UV-Visible spectrophotometer, Agilent, Santa Clara, CA, USA, and UV-1280 UV-Vis spectrophotometer, Shimadzu, Kyoto, Japan). The Dox content was determined by comparing with the standard curve of Dox in methanol, and used for determining the percentage of loading capacity (%LC) and entrapment efficacy (%EE) of Dox in micelles, according to the following formulation:

(1) $$\begin{array}{cc} \%\;\mathrm{LC}= & \frac{\mathrm{Amount}\;\mathrm{of}\;\mathrm{doxorubicin}\;\mathrm{in}\;\mathrm{nanoparticles}}{\mathrm{Amount}\;\mathrm{of}\;\mathrm{nanoparticles}} \end{array}\;\times \;100$$

(2) $$\begin{array}{cc} \%\;\mathrm{EE}= & \frac{\mathrm{Measured}\;\mathrm{amount}\;\mathrm{of}\;\mathrm{doxorubicin}\;\mathrm{in}\;\mathrm{nanoparticles}}{\mathrm{Total}\;\mathrm{amount}\;\mathrm{of}\;\mathrm{doxorubicin}\;\mathrm{used}} \end{array}\;\times \;100$$

### 2.5. Study of Interaction between Dox-HCl and P407 of DMs by Assessing Crystallinity and Thermal Behavior

To investigate the status of encapsulated Dox in P407-micelles, crystalline characteristics of Dox-micelles were analyzed. DM and blank-micelle (BM) solutions were frozen at −80 °C overnight and lyophilized to obtain micelle powder form. The lyophilized DMs and BMs, Dox-HCl powder, and P407 were analyzed for crystallinity using a Rigaku Smartlab powder diffractometer (XRD) (Rigaku, Tokyo, Japan) with the scattering angle of 2θ from 0 to 60° at room temperature, and data was analyzed using Origin software. For thermal behavior, 5 mg of lyophilized micelles, Dox-HCl powder, and P407 were determined at a heated temperature ranging from 0 to 300 °C with a linear heating rate of 5 °C/min using a TA Instruments Q200 Differential Scanning Calorimeter (DSC) (TA Instruments, New Castle, DE, USA), and data were analyzed using TA universal software (universal analysis 2000 for Windows 2000/XP/Vista), version 4.5A.

### 2.6. In Vitro Dox Release

A dialysis method was used to perform Dox release from encapsulated micelles. DMs and Dox-HCl solutions were placed in a 3.5 kDa MWCO dialysis bag and incubated in 30 mL of PBS, pH 7.4, containing 0.5% *w*/*w* tween 80 in light-protecting containers. For 72 h, each dialysis bag was continuously swirled at 37 °C and 100 rpm. Release media (1 mL) was collected at each chosen time interval of 0, 1, 2, 4, 6, 8, 10, 12, 24, 48, and 72 h, and the same volume of fresh medium was added to replace the withdrawal. The amount of Dox in each time-collected sample was measured using a UV-Vis spectrophotometer or HPLC in the conditions described in the previous section and calculated by comparing with a standard curve.

### 2.7. Stability of DMs

The colloidal stability of DMs was evaluated by determining the changes in particle size, PdI, and zeta potential after incubating with 1× PBS, pH 7.4 containing 1%, 5%, and 10% BSA for 24 h, comparing with freshly prepared samples. In addition, to study the effects of time and temperature on DM stability, the micelles were stored at 4 °C, RT, and −80 °C for 30 days in light-protecting containers, and then the changes in particle size, PdI, zeta potential, %EE, and %LC of DMs were evaluated on days 1, 3, 5, 10, 15, 20, and 30 at RT and compared with a standard curve.

### 2.8. Synthesis and Characterization of P407-FLT3 Peptide Conjugation

#### 2.8.1. Cargo Azide Preparation and Characterization

P407 was firstly modified before conjugating with FLT3 peptides. The terminal hydroxy group (-OH) of the P407 poloxamer was converted to an aldehyde group (-CHO) by dissolving 1 g of P407 in 30 mL of anhydrous dichloromethane (DCM), followed by adding 58.1 g of Dess–Martin periodinane (DMP) into P407-DCM solution under stirring at 400 rpm, RT, for 24 h. Following incubation, the mixture was added into cold petroleum ether (350 mL) and incubated at −20 °C for 24 h to precipitate P407-CHO. Finally, the cold petroleum ether was poured out and the precipitate was dried under nitrogen gas. Afterwards, dried P407-CHO was used as substrate to synthesize “cargo azide” (CA), which is P407 with functional azide. Briefly, 1 g of P407-CHO was dissolved in 100 mL of THF, then 13.89 mg of amino-PEG6-azide and 17 mg of STAB were added to P407-CHO solution while stirring and reacted for 24 h at RT under a nitrogen atmosphere. After that, 0.324 mL of methanol was added to the mixture to quench the remaining STAB reaction. The mixture was stirred for 1 h at RT, and then the THF was removed from the cargo azide product by solvent evaporation at reduced pressure. The CA was air dried at RT overnight, followed by dilution in water and purification using Amicon^®^ Ultra 15 mL Centrifugal Filters 4.5 kDa MWCO three times to remove unconjugated amino-PEG6-azide. To confirm the success of the modification process, each sample was dissolved in chloroform-D (CDCl_3_) at a concentration of 20–30 mg/600 µL and evaluated for the change in ^1^H and ^13^C-NMR curve.

#### 2.8.2. Cargo Azide-FLT3 Peptides Conjugation and Characterization

In order to synthesize polymer conjugated synthesized FLT3-peptide, alkyne-modified peptides, CKR1 and EVQ, were attached to CA by a copper-catalyzed azide-alkyne cycloaddition (CuAAC) reaction (Click chemistry) as described in Presolski, 2011 [40]. In brief, the stock reagent, including 248.45 µM of peptide-alkyne in potassium phosphate (KH_2_PO_4_) buffer, 5 mM of CA in KH_2_PO_4_ buffer, a pre-mixed solution of 20 mM of CuSO_4_ and 50 mM of ligand THPTA in water, 5 mM of aminoguanidine hydrochloride in water, and 100 mM of L-sodium ascorbate in water, were combined to obtain the final concentration of peptide-alkyne at 200 µM, CA at 400 µM, CuSO_4_ at 100 µM, ligand THPTA at 500 µM, aminoguanidine hydrochloride at 5 mM, and L-sodium ascorbate at 5 mM in the 25 mL reaction. The mixture was inverted using a rotisserie tube rotator or orbital shaker from at least 1 h to overnight, then filtrated through Amicon^®^ Ultra 15 mL Centrifugal Filters, 4.5 kDa MWCO, to collect the conjugates. The conjugates were lyophilized to obtain the CA-peptide in powder form. All modified polymers were kept at −20 °C until use. To confirm the success of conjugation, ^1^H-NMR and UV-vis absorption spectra of P407 and CA-peptide conjugates were compared using a UV-Vis Spectrophotometer.

#### 2.8.3. Fourier Transform Infrared (FTIR)

The interaction of CA and FLT3 peptide was confirmed using FTIR analysis. FTIR spectra of samples were collected using FTIR spectroscopy (470FT-IR, Nicolet Nexus, Waltham, MA, USA) at a resolution of 64 cm^−1^ in range of 500–4000 cm^−1^.

### 2.9. DMs Conjugated with FLT3 Peptides: Preparation and Physiochemical Assessment

Several formulations of DM-conjugated FLT3 peptides comprised of DM-CKR, DM-EVQ, and DM-CKR+DM-EVQ were prepared by using Dox-HCl and modified polymers at a weight ratio of Dox-HCl to polymer at 1:200 mg. Initially, 40 mg of CA-CKR and CA-EVQ were used to form BM-CKR and BM-EVQ, whereas 20 mg of CA-CKR were combined with an equivalent amount of CA-EVQ to form mixed-micelle BM-CKR+BM-EVQ. The modified polymers were dissolved in methanol at a concentration of 1 mg/mL, and then evaporated under reduced pressure in a rotary evaporator and dried at RT overnight. The micelle-films were rehydrated in 2.8 mL of NSS, followed by 0.4 mL of 10× PBS, pH 7.4, and 0.2 mg Dox-HCl in 0.8 mL solution while stirring to produce micelles as described in Section 2.3.

After that, particle size, polydispersity (PdI), zeta potential (ZP), %EE, and %LC of fresh DMs conjugated with peptides were determined in accordance with Section 2.4.

### 2.10. Investigation of DMs Conjugated with FLT3 Peptides Stability

DM-CKR, DM-EVQ, and DM-CKR+DM-EVQ were incubated with 1× PBS, pH 7.4 containing 10% BSA for 24 h, and then particle size, PdI, and zeta potential were evaluated to assess the colloidal stability. Furthermore, the micelles were stored at −80 °C for 60 days in light-protecting containers to study the changes in particle size, PdI, zeta potential, %EE, and %LC at RT.

### 2.11. Assessment of Cytotoxic Effects of Dox-HCl Solution and DMs with or without FLT3 Peptides on AML Leukemic Cells

The MTT assay was performed to determine cytotoxic effects of Dox in solution and micelle form. KG-1a (1.5 × 10^4^ cells/100 µL) and EoL-1 (3 × 10^4^ cells/µL) cells were plated into flat-bottom 96-well plates for 24 h. Dox-HCl solution, DM, DM-CKR, DM-EVQ, and DM-CKR+DM-EVQ were prepared in a 2-fold dilution at concentrations of 0–4 µg/mL in 100 µL and incubated with seeded cells at 37 °C, 5% CO_2_ atmosphere for 48 h (final concentrations were 0–2 µg/mL). After that, 100 µL of treatment medium was discarded, and 15 µL of MTT solution (1 mg/mL) was added and further incubated for 4 h. After removing the supernatant, 200 µL of DMSO was added to dissolve the formazan crystals. The optical density was measured at 578 nm with the reference wavelength at 630 nm using a SpectraMax^®^ M5 Microplate Reader (Molecular Devices, San Jose, CA, USA). The percentage of viable cells was calculated from the absorbance of test and control samples using Equation (3).

(3) $$\begin{array}{cc} \%\;\mathrm{Cell}\;\mathrm{viability}= & \frac{\mathrm{Mean}\;\mathrm{absorbance}\;\mathrm{in}\;\mathrm{test}\;\mathrm{well}}{\mathrm{Mean}\;\mathrm{absorbance}\;\mathrm{in}\;\mathrm{vehicle}\;\mathrm{control}\;\mathrm{well}} \end{array}\;\times \;100$$

### 2.12. Monitoring of Cellular Uptake of Dox-HCl Solution and DM Conjugated with and without Peptides In Vitro Using a Fluorescence Microscope

KG-1a cells (3 × 10^5^ cells/mL) were incubated with Dox-HCl solution, DM, DM-CKR, DM-EVQ, and DM-CKR+DM-EVQ at concentrations of 3 µg/mL at 37 °C, 5% CO_2_ atmosphere for 5 h. Following incubation, treated cells were collected and washed twice with ice-cold PBS, pH 7.4, using a microcentrifuge at 5000 rpm for 10–15 s, and smeared on a glass slide and air dried for 15–30 min. Dry smears were fixed using 1 mL of cold methanol for 1 min, followed by 1 mL of cold acetone for 1 min after the methanol was removed. After drying, the cell nucleus was stained by adding 500 µL of 300 nM of DAPI for 10 min in a light-protecting container, and the smears were rinsed twice with ice-cold PBS, pH 7.4, for 2 min each time, and 20 µL PBS, pH 7.4, was added to the smears and then covered with coverslips. The fixed cells were observed under a fluorescence microscope (Zeiss, Jena, Germany) and analyzed using Zen software (ZEN 2.6, blue edition). The fluorescence of Dox observed in Dox-micelle-treated samples was qualitatively compared with Dox-HCl-solution-treated samples.

### 2.13. Determination of Dox-HCl Solution and DM Conjugated with and without Peptides on Apoptosis Induction

To investigate the effect of several formulations of DMs on cell apoptosis induction, flow cytometry was performed by using an FITC Annexin V Apoptosis Detection Kit with PI. KG-1a cells (1.5 × 10^5^ cells/mL) were incubated with Dox-HCl solution, DM, DM-CKR, DM-EVQ, and DM-CKR+DM-EVQ at concentrations of 0.8 µg/mL at 37 °C, 5% CO_2_ atmosphere for 48 h. After that the treated cells were collected and washed twice with cold PBS, pH 7.4, and resuspended in 100 µL Annexin V Binding Buffer in FAC tube, then 5 µL of FITC Annexin V and 10 µL of Propidium Iodide (PI) solution was added. The cells were gently vortexed and incubated for 15 min at RT in the dark. After incubation, 400 µL of Annexin V Binding Buffer was added to the cell suspension and analyzed using a flow cytometer (Cytomics FC500 flow cytometer, Beckman Coulter, Brea, CA, USA).

### 2.14. Investigation of the Effects of FLT3 Peptides on FLT3 Protein Expression

KG-1a cells (1.5 × 10^5^ cells/mL) were treated with Blank-micelle (BM), BM-CKR, BM-EVQ, and BM-CKR+BM-EVQ at concentrations of 0.3 µg/mL at 37 °C, 5% CO_2_ atmosphere for 48 h. The treated cells were harvested and washed twice with cold PBS, pH 7.4 after incubation. The total proteins were extracted in RIPA buffer and the concentration was determined using the Folin–Lowry method. The protein lysates were separated using 12% SDS-PAGE and transferred to PVDF membranes. The membrane was cut to separate FLT3 (target protein) and GAPDH (internal control protein), blocked in 5% skim milk, and then probed with rabbit polyclonal anti-FLT3 antibody at a dilution of 1:1000 and rabbit polyclonal anti-GAPDH antibody at a dilution of 1:10,000. Afterwards, a 1:15,000 dilution of HRP-conjugated goat anti-rabbit IgG was added to the reaction. Luminata^®^ Forte Western HRP substrate was used to visualize the proteins. The protein band signal (chemiluminescence) was detected using X-ray film and quantified with a scan densitometer (Bio-Rad, Hercules, CA, USA).

### 2.15. Statistical Analysis

All data were presented as mean ± standard deviation (SD) or mean ± standard error of mean (SEM) from three independent experiments. The statistical differences between samples were determined using a one-way ANOVA. At a *p*-value < 0.05, the data was considered statistically significant.

## 3. Results

### 3.1. Formula Optimization and Characterization of DM

To establish an optimal formulation for DM preparation, several formulations of DM were prepared by adjusting the weight ratio of Dox-HCl to P407 from 1:40 to 1:240 mg (Table 1). BMs were firstly prepared with P407 using the film-hydration technique, and then Dox-HCl was loaded in BM via the pH-induced self-assembly method. Physiochemical characteristics of each DM formulation were determined. The results showed that the particle sizes of all freshly prepared DM formulations ranged from 22 to 28 nm with a narrow distribution (PdI < 0.300). The zeta potential demonstrated that formulations had negative surface charges of about 0 to −5 mV. The %EE of Dox in each micelle was found to be higher than 90%. This result demonstrated the success of Dox-loading poloxamer micelle preparation. After five days of incubation at RT, only DM8, DM9, and DM10 exhibited particle sizes less than 60 nm and %EE higher than 90% (data not shown). Other formulations, on the other hand, had mean particle sizes greater than 100 nm, which could be related to micelle aggregation. However, DM8 showed a good particle size and drug encapsulating efficacy in parallel with DM9 and DM10. Furthermore, it used a smaller amount of P407 for core-shell micelle synthesis, leading to a higher %LC in DM8.

**Figure 1:**
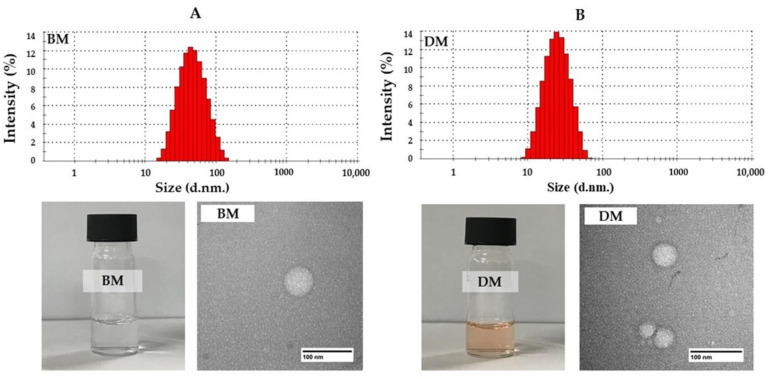
Physical appearance and particle size distribution histogram from DLS, and morphology from TEM of (**A**) blank micelles (BMs) and (**B**) Dox-micelles (DMs).

As a result, freshly prepared DMs with a weight ratio of Dox and P407 of 1:200 mg (DM8) exhibited particle sizes averaging 23.33 ± 1.25 nm, PdI of 0.15 ± 0.02, zeta potential of −1.41 ± 2.09 mV, with %EE and %LC of 92.85 ± 1.67% and 0.44 ± 0.1%, respectively. The result corresponded to the red-clear appearance of the micelle solution, suggesting the optimal formulation for the Dox-loaded micelle preparation (Figure 1A). The particle sizes, PdI, and zeta potential of BMs were 23.58 ± 1.10 nm, 0.11 ± 0.01, and −0.44 ± 0.63 mV, respectively, which were close to DMs, indicating that Dox loaded into micelles did not affect the size and charge of micelles (Figure 1B). The morphology of BM and DM at a weight ratio of 1:200 was determined using TEM. The spherical nanoparticles with a size under 100 nm were observed from the samples. The particle size after TEM correlated with the results obtained from dynamic light scattering using a Zetasizer (Figure 1A,B).

### 3.2. Interaction between Dox-HCl and P407 in DM

**Figure 2:**
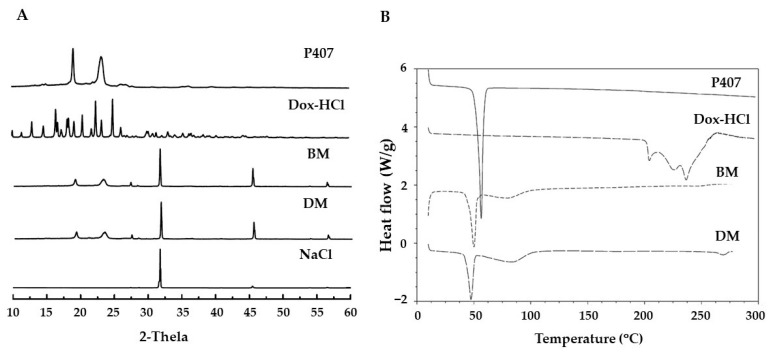
(**A**) Crystalline characteristics and (**B**) thermal behavior of Dox in Dox-micelle (DM) compared with blank-micelle (BM), Dox-HCl, and P407.

To determine entrapment of Dox in poloxamer micelles, XRD and DSC analyses were performed. The natural crystalline pattern of Dox from XRD exhibited strong peaks at scattering angles ranging from 13° to 25°, whereas the diffraction peaks at 19° and 23° were detected in P407 and BM. In the DM sample, only the typical peaks of P407 were seen, indicating that Dox was in the amorphous form within the micelles. Both BM and DM found a new peak at 27°, these new peaks were probably seen due to the arrangement of P407 molecules in the micelle formation process. In addition, the diffraction peaks of NaCl in PBS, pH 7.4 were seen at 33°, 46°, and 55° due to the use of PBS, pH 7.4, which induced Dox incorporation into the core-micelle during Dox-micelle formation. Overlaid XRD patterns of P407, Dox, lyophilized BM, lyophilized DM, and NaCl were shown in Figure 2A. Moreover, DSC was utilized to investigate the thermal behavior of DM to confirm drug loading. The DSC thermogram showed an endemic melting peak of P407 at 56.67 °C, and three melting peaks of Dox at 204.03, 225.99, and 236.96 °C. Meanwhile, the thermogram of DM showed all endothermic peaks of polymers as shown in the BM thermogram, but the characteristic peaks of Dox were not observed, which confirmed the encapsulation of Dox inside the micelle in amorphous state. However, there was a slight decrease in melting point and the presence of a broadening peak (80–90 °C) that differed from P407. This phenomenon could be attributed to the new crystalline structure that occurred during the formation of P407 micelles. The melting patterns of DM, BM, Dox-HCl, and P407 are shown in Figure 2B.

### 3.3. In Vitro Release Profile of DM

**Figure 3:**
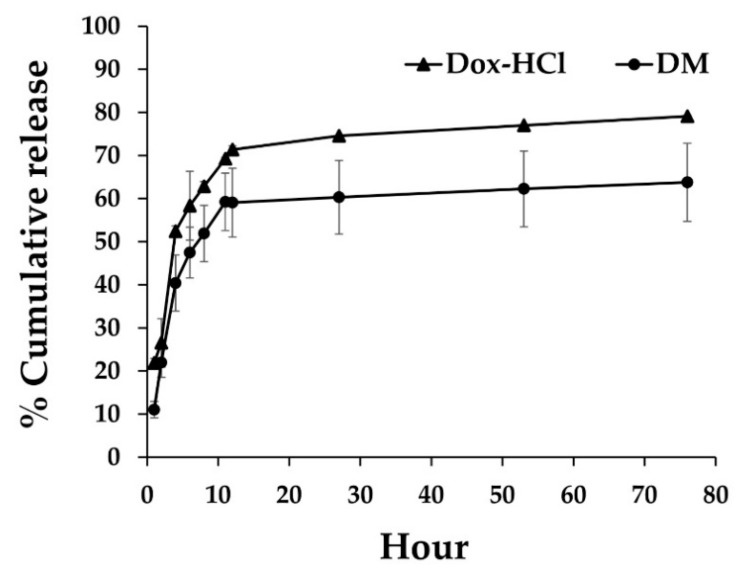
Drug release profiles of Dox from DM and Dox-HCl solution under pH 7.4. Data were presented as mean ± SD (*n*  =  3).

The in vitro Dox-releasing capacity of DMs and Dox-HCl in PBS, pH 7.4, that corresponds to the extracellular pH reported in cancer patients, was evaluated. As shown in Figure 3, the cumulative release profiles at 24 h of incubation time of Dox in solution and polymeric micelles were approximately 75% and 60%, respectively. The release of Dox in aqueous form was appeared to be significantly higher than Dox from DM, suggesting that the P407 polymeric micelle had the ability to encapsulate Dox within the hydrophobic core of micelle and may extend the drug release time.

### 3.4. Effects of Colloid, Temperature, and Time on DM Stability

**Figure 4:**
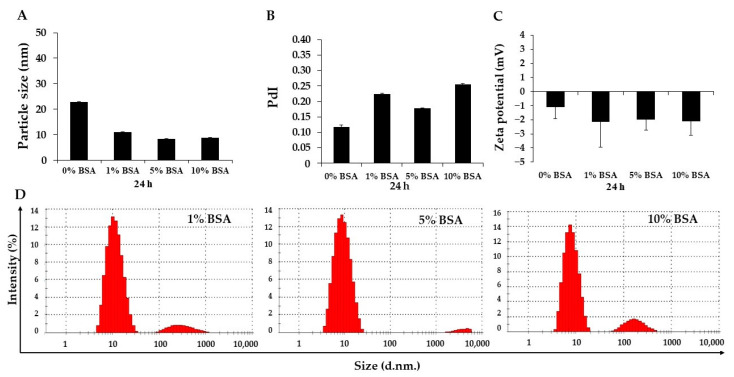
The alterations in (**A**) particle size, (**B**) polydispersity index (PdI), (**C**) zeta potential value, and (**D**) particle size distribution histogram of Dox-micelle (DM) after 24 h of incubation in PBS, pH 7.4 containing 0%, 1%, 5%, and 10% BSA, respectively, at 37 °C in the dark. The data were shown as mean ± SE (*n* = 3).

In order to assess the colloid stability of DMs, the particle size and zeta potential of micelles in various concentrations of bovine serum albumin (BSA) solution were investigated. Following 24 h of incubation time, the mean particle sizes of DMs dissolved in PBS, pH 7.4 with 1%, 5%, and 10% BSA were reduced to 11.20 ± 0.08, 8.32 ± 0.12, and 8.81 ± 0.12 nm, respectively, which were significantly different from the initial size of DMs in NSS-PBS solution (22.92 ± 0.07 nm), while the PdI values slightly increased (Figure 4A,B). The previous report demonstrated that an average particle size should be around 8 nm in solution [41]. According to the size distribution histogram, 90% of the DMs in 1% and 10% BSA solutions ranged from 5 to 20 nm and 10% were 100–1000 nm. Meanwhile, DM in 5% BSA solution exhibited 97% of particle sizes ranging from 5 to 20 nm, suggesting that excluding BSA made the particle size similar to DM in the 0% BSA solution. However, some presented an aggregated form, resulting in large particle sizes and increased PdI value (Figure 4D). The zeta potential of the micelles was more negative as a result of the increase in %BSA (Figure 4C). Moreover, although the concentration of BSA was increased, the size and zeta potential of DM in each BSA-PBS solution were quite similar. The hydrophilic PEO block is known to prevent the adsorption of albumin on the micellar surface [32]. It was feasible this may prolong the circulation of micelles under physiological conditions.

**Figure 5:**
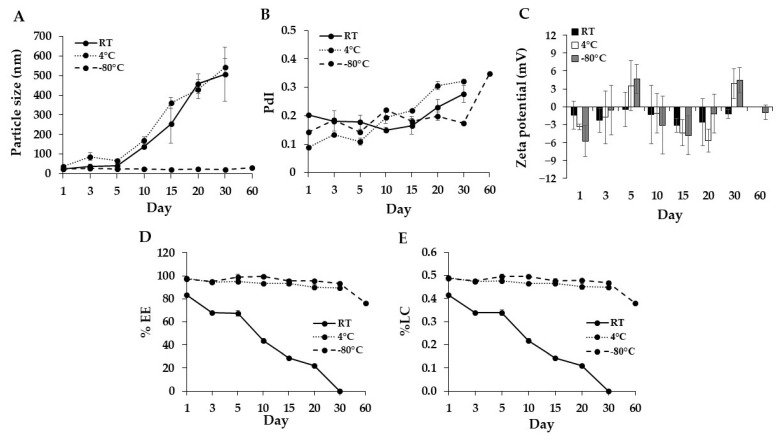
The variations in particle size (**A**), polydispersity index (PdI) (**B**), zeta potential (**C**), %EE of Dox (**D**), and %LC of Dox in Dox-micelle (**E**) after 30 and 60 days of storage at room temperature (RT), 4 °C, and −80 °C in the dark, respectively. Size, PdI, zeta potential, % EE, and % LC data were presented as mean ± SE (*n* = 3).

Additionally, to evaluate the stability of DMs under different storage conditions, DMs were stored at 4 °C, RT, and −80 °C in the dark for 2 months and their physiochemical properties were examined. The results showed that DMs stored at −80 °C could retain physical properties comparable with fresh samples (particle size < 30 nm). In contrast, at RT and 4 °C of storage, the size of DM increased to over 250 nm after 10 days of storage (Figure 5A), suggesting that increasing temperature probably caused micelle aggregation. PdI and zeta potential of micelles were also changed after long-term storage, although no significant difference was seen between temperatures. The possibility of particle agglomeration increased in all storage conditions, whereas the zeta potential remained within the neutral range (Figure 5B,C). The %EE and %LC of Dox in DM stored at 4 and −80 °C were approximately 90% and 0.4%, respectively, which was close to newly prepared samples. On the other hand, storage at RT showed a gradual decrease in Dox content (Figure 5D,E). Furthermore, the physiochemical property of DMs was checked after storage at −80 °C for 2 months. The results showed that micelles could maintain their size under 30 nm, although PdI increased slightly, and %EE decreased to 76%. In this study, a temperature of −80 °C was the optimal temperature for maintaining the physiochemical stability of micelles.

### 3.5. Synthesis and Characterization of P407-FLT3 Peptide Conjugation

**Figure 6:**
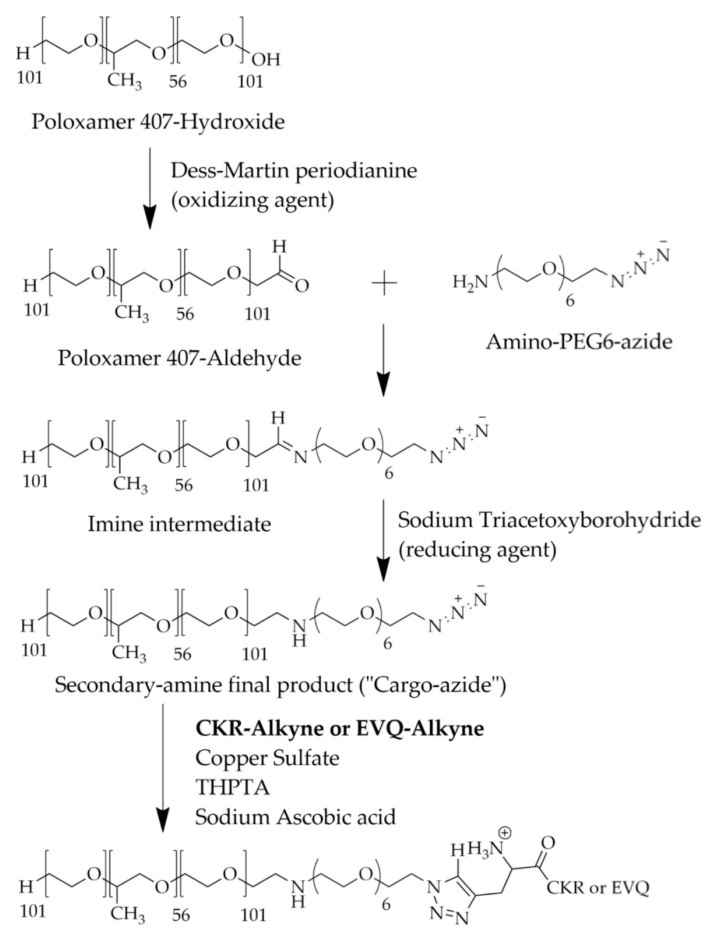
Scheme of cargo azide (CA) and CA conjugated peptide synthesis.

**Figure 7:**
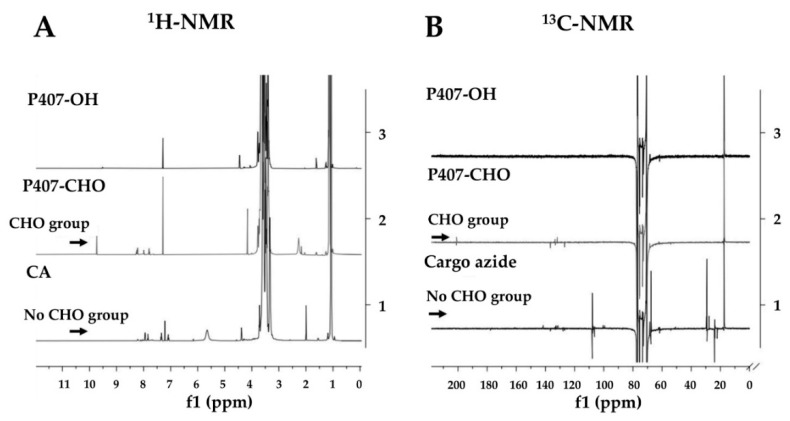
Characterization of cargo azide (CA). (**A**) ^1^H-NMR and (**B**) ^13^C-NMR spectrum of P407-OH, P407-CHO, and CA.

The scheme of cargo azide synthesis and cargo azide-peptide conjugation is shown in Figure 6. To prepare micelle conjugated peptides, P407 was firstly modified and conjugated with peptides. For P407 modification, the hydroxyl (OH) group at both ends of the P407 poloxamer (P407-OH) was firstly converted to the aldehyde (CHO) group (P407-CHO) using Dess–Martin periodinane (DMP), that oxidized primary alcohols to aldehydes. The complete reaction was confirmed by a CHO-group peak at δ = 9.75 ppm in the ^1^H-NMR spectrum and δ = 200 ppm in ^13^C-NMR. Then, P407-CHO was used as a substrate for cargo azide (CA), azide functionalization of P407, and synthesis by coupling to the azido-PEG-amine linker. In this study, the reducing agent, sodium triacetoxyborohydride, was added to the reaction to prevent intermediated imine production, which could affect CA stability. The absence of a CHO-peak in ^1^H- and ^13^C-NMR demonstrated that P407 and linker had been completely conjugated. The NMR spectra of P407-OH, P407-CHO, and cargo-azide are shown in Figure 7A,B.

**Figure 8:**
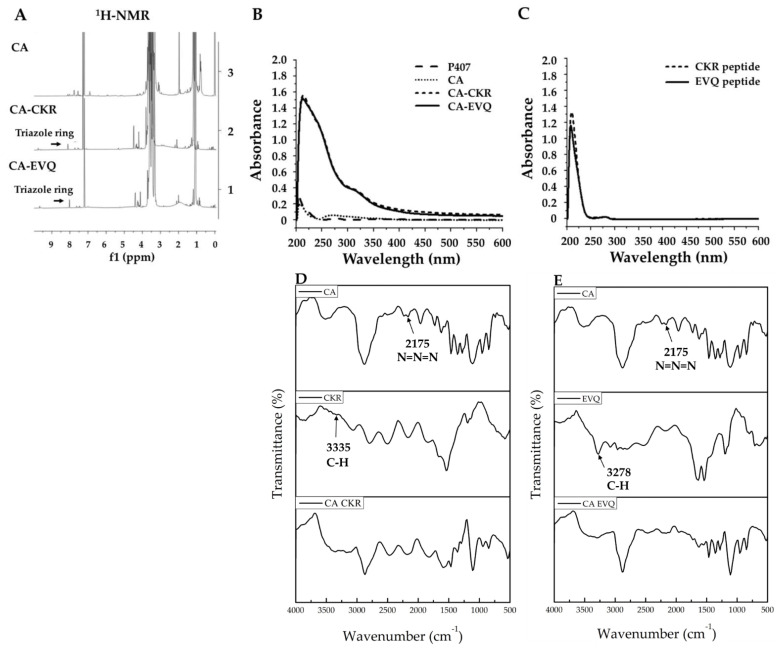
Characterization of cargo azide conjugated FLT3 peptides, CKR (CA-CKR) and EVQ (CA-EVQ). (**A**) ^1^H-NMR spectrum of CA, CA-CKR, and CA-EVQ, (**B**) UV-Vis spectra of P407, CA, CA-CKR, and CA-EVQ, and (**C**) UV-Vis spectra of CKR and EVQ peptides. (**D**) FTIR spectra of CA, CKR peptides, CA-CKR, and (**E**) FTIR spectra of CA, EVQ peptides, and CA-EVQ.

Following CA and specific alkyne-modified FLT3 specific peptides, CKR and EVQ were conjugated using the copper-catalyzed azide-alkyne cycloaddition (CuAAC) reaction to produce polymer conjugated peptides. The ^1^H-NMR spectrum of cargo azide conjugated CKR or EVQ peptides (CA-CKR and CA-EVQ) demonstrated a new triazole ring peak at 8.02–8.12 ppm (Figure 8A). Moreover, the successful CA-peptide conjugation was also established using UV-Vis spectrometry. The results were approved by the strong absorption peak of peptides at 210 nm in conjugation samples compared with CA, P407, and unconjugated peptides (Figure 8B,C). The FTIR spectra of cargo azide (CA), FLT3 peptide CKR and EVQ, and CA conjugated FLT3 peptide are shown in Figure 8D,E. The IR spectrum at 2175 cm^−1^ corresponded to N=N=N stretching vibration in amino-PEG-azide linker coupled with P407-CHO to form CA. Meanwhile, EVQ and CKR peptides demonstrated C-H stretching vibration at 3278 and 3335 cm^−1^ of the alkyne group in alkyne-EVQ and CKR peptides, respectively. After CA was conjugated with peptides, the N=N=N stretching vibration of CA and the C-H stretching vibration in the alkyne of EVQ and CKR peptides disappeared suggesting that the alkyne group of peptides could attach to the azide group of CA. This result confirmed the effective synthesis of CA-peptide. However, the NMR-observed triazole ring was not detected in the IR spectra. In a previous study, the OEG peptide was clicked in situ with propargylamine modified Au nanoparticles (PA-AuNPs) to form triazole-linked Au nanoparticles (OEG-Au NPs) [42]. The presence of triazole bands at 1636 and 1436 cm^−1^ in the IR spectrum of OEG-Au NPs indicates that the click reaction was successful. In our work, the triazole might be masked by the C=O stretching vibration (1460 cm^−1^) in both CKR and EVQ peptides, which is extremely close to the wavelength of the triazole. Therefore, the peaks were undetectable.

### 3.6. Preparation and Characterization of DM-CKR, DM-EVQ, and DM-CKR+DM-EVQ

**Figure 9:**
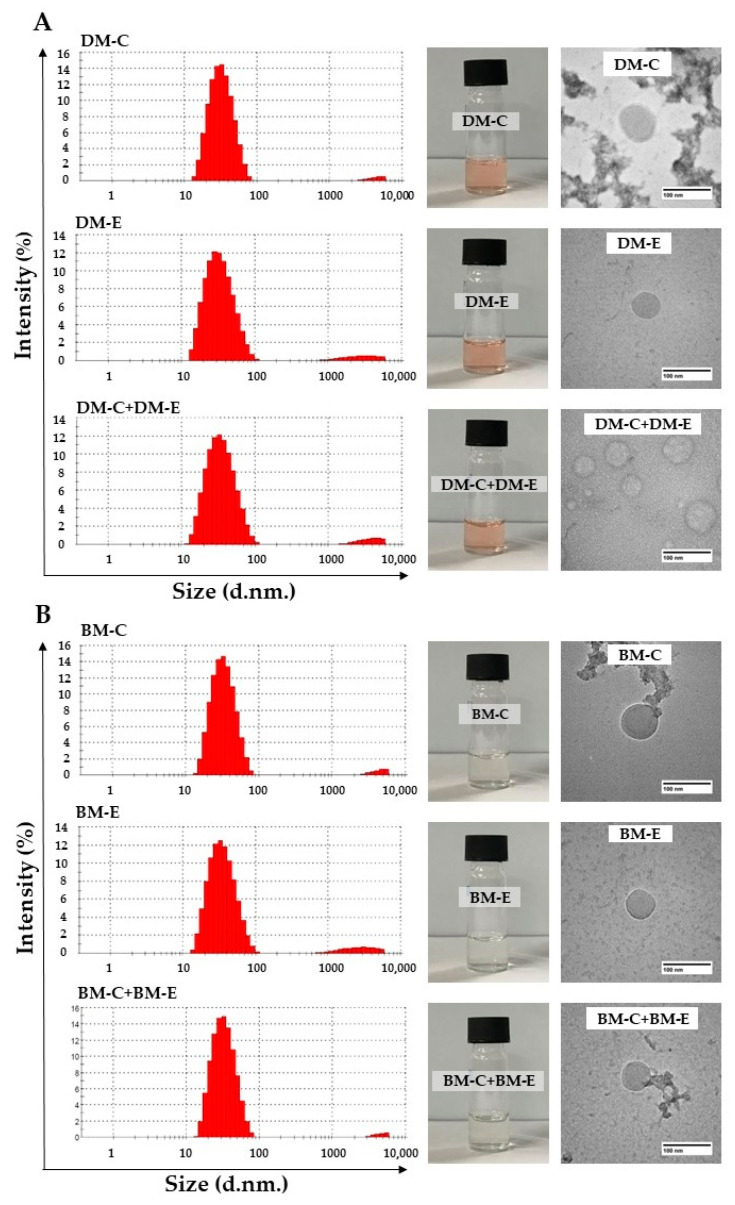
Particle size distribution histogram using DLS, physical appearance, and morphology from TEM of (**A**) BM conjugated FLT3 peptides (BM-CKR, BM-EVQ, and BM-CKR+BM-EVQ) and (**B**) DM conjugated FLT3 peptides (DM-CKR, DM-EVQ, and DM-CKR+DM-EVQ).

Following the polymer–peptide conjugation step, blank and Dox-micelle conjugated with peptides were produced using film-hydration and pH-induced self-assembly method at a weight ratio of Dox to polymer at 1:200. The micelles included DM, Dox encapsulated in micelle conjugated CKR (DM-CKR), Dox encapsulated in micelle conjugated EVQ (DM-EVQ), and Dox encapsulated in mixed-micelle formulated from CA conjugated CKR and CA conjugated EVQ (DM-CKR+DM-EVQ). After centrifugation, a red-clear DM solution was obtained. The freshly synthesized DM-CKR, DM-EVQ, and DM-CKR+DM-EVQ showed a narrow particle size dispersion ranging from 34 to 45 nm and had a low zeta potential of −1 to −3 mV. In addition, the %LC and %EE DM conjugated with peptides were approximately 0.4% and 70%, respectively, as shown in Table 2. The addition of peptides to the modified polymer probably affected micelle formation, resulting in an increase in micelle size and a decrease in drug loading in comparison with DM without peptides. Nonetheless, the drug content in the micelles was greater than 70%, indicating that Dox could be well packaged in the core-shell micelles. The TEM observation revealed that the spherical nanoparticle size of each DM coupled peptide was less than 100 nm, which was comparable to the size distribution histogram data. However, particle sizes higher than 1000 nm were also observed, which might be caused by micelle aggregation. The mean size distribution, physical appearance, and morphology of BM and DM conjugated FLT3 peptides are shown in Figure 9A,B.

### 3.7. Effects of Colloid, Temperature, and Time on DM Conjugated Peptides’ Stability

**Figure 10:**
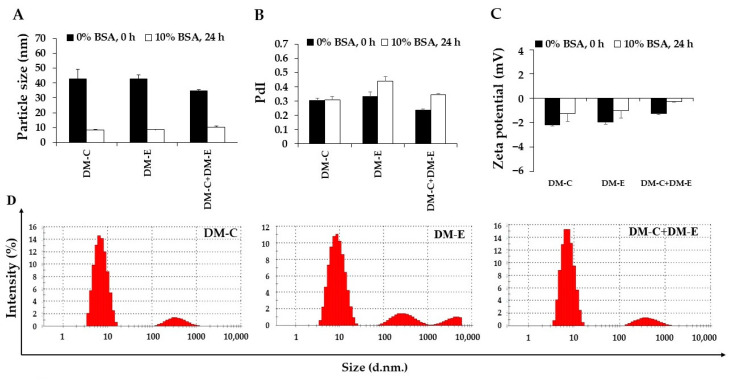
The alterations in (**A**) particle size, (**B**) polydispersity index (PdI), (**C**) zeta potential value, and (**D**) particle size distribution of Dox-micelle conjugated FLT3 peptides, including DM-CKR (DM-C), DM-EVQ (DM-E), and DM-CKR+DM-EVQ (DM-C+DM-E) after 24 h of incubation in PBS, pH 7.4 containing 10% BSA at 37 °C in the dark. The data were shown as mean ± SE (*n* = 3).

Since there was no difference in size or zeta potential in the study of colloidal stability of DM in different concentrations of BSA in PBS solution, DM-CKR, DM-EVQ, and DM-CKR+DM-EVQ were incubated for 24 h in PBS containing 10% BSA, which was close to physiological conditions. The results showed that the mean particle size of all samples was reduced to 8–10 nm, which was half the original micelle size found in DM. This could be mostly attributed to the size of BSA. However, large particle sizes (particles in the range of 100–1000 nm) were observed in about 15% of the total particles from the size distribution, indicating that some particle aggregation occurred, which is also supported by the increase in PdI value (Figure 10A,B). The zeta potential of each formulation appeared to be negative or close to neutral (Figure 10C).

Furthermore, the physiochemical properties of DM with peptides were assessed after 60 days of storage at −80 °C, which is the optimal storage temperature for DM. On day 60, all DM conjugated peptides exhibited an increase in particle size and PdI ranging from 40 to 50 nm and 0.4–0.5, respectively, while DM tended to remain steady (data are shown in Section 3.4). The increased size and PdI of DM with peptides were most likely caused by the addition of peptides, which may have had an effect on the original micelle formation. In terms of Dox content, the results demonstrated a decrease in %EE of Dox in all DM formulations, especially DM-EVQ. Storage at −80 °C is the most appropriate condition for preserving both DM with and without peptides (Table 3).

### 3.8. Determination of Cytotoxic Effects of Dox-HCl, DM, and DM Conjugated with FLT3 Specific Peptides on Leukemic Cell Viability

**Figure 11:**
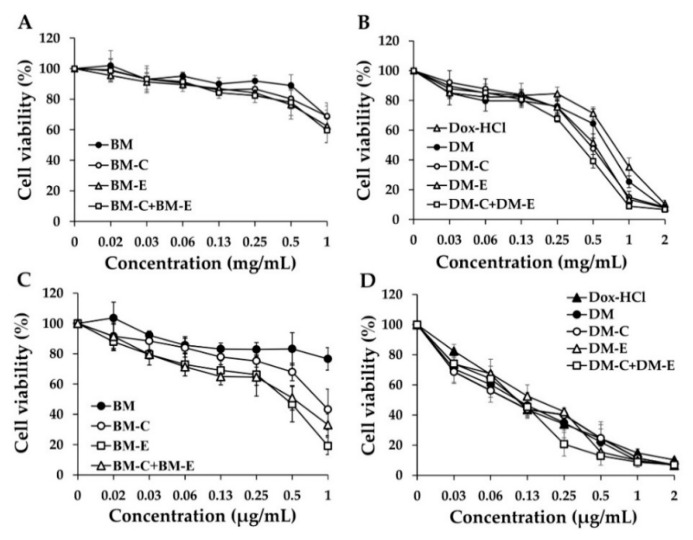
Cytotoxicity of blank-micelle (BM), BM-CKR (BM-C), BM-EVQ (BM-E), and BM-CKR+BM-EVQ (BM-C+BM-E) against (**A**) KG-1a and (**B**) EoL-1 cells. Cytotoxic effects of Dox-HCl, DM, DM-CKR (DM-C), DM-EVQ, and DM-CKR+DM-EVQ (DM-C+DM-E) against (**C**) KG1a and (**D**) EoL-1 cells. The data were shown as mean ± SD (*n* = 3).

In this experiment, KG-1a and EoL-1 cells (FLT3-positive AML cells) were treated with Dox-HCl and several formulations of DMs at equivalent Dox doses of 0–2 µg/mL and their empty micelles at concentrations of 0–1 mg/mL for 48 h. Cytotoxicity was evaluated using an MTT assay. According to the cell viability curve, all formulations of blank-micelles and BMs with conjugated peptides did not affect cell viability in KG-1a and EoL-1 cells at the final concentration (1 mg/mL), while BM-CKR, BM-EVQ, and BM-CKR+BM-EVQ showed cytotoxicity against EoL-1 cells with IC_50_ values of 0.89 ± 0.19, 0.47 ± 0.15, and 0.49 ± 0.19 mg/mL, respectively (Figure 11A,B). DM, DM conjugated with one peptide (DM-CKR and DM-EVQ), and DM with a combination of peptides (DM-CKR+DM-EVQ) could increase the cytotoxicity of Dox towards KG-1a cells compared with Dox aqueous solution (Figure 11C). In KG-1a cells, IC_50_ values for the Dox-HCl solution, DM, DM-CKR, DM-EVQ, and DM-CKR+DM-EVQ were 0.80 ± 0.06, 0.68 ± 0.06, 0.49 ± 0.09, 0.52 ± 0.04, and 0.41 ± 0.03 µg/mL, respectively. The results demonstrated that the IC_50_ value of DM was statistically significantly lower than the Dox solution. All DM conjugated peptides were also considerably lower than both DM and Dox solution. Interestingly, DM-CKR+DM-EVQ showed the highest toxicity compared with other DM formulations. In contrast, the cytotoxic activity of Dox in solution and micelle form showed a similar trend in EoL-1 cells (Figure 11D). The IC_50_ values for Dox-HCl solution, DM, DM-CKR, DM-EVQ, and DM-CKR+DM-EVQ in EoL-1 cells were 0.11 ± 0.01, 0.11 ± 0.04, 0.10 ± 0.03, 0.16 ± 0.04, and 0.11 ± 0.03 µg/mL, respectively. The EoL-1 cells are known to be sensitive to Dox-HCl treatment. Thus, there was possibly no difference between the toxicity of solution and micelle forms of Dox. Accordingly, AML leukemic stem-cell-like KG-1a cells which are noted to be resistant to Dox, were chosen for further study.

### 3.9. Apoptosis Induction of Dox-HCl and DM Formulations

**Figure 12:**
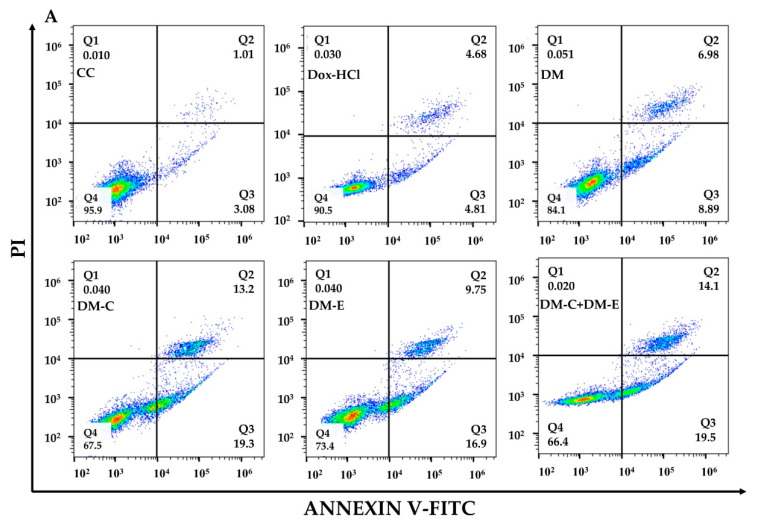

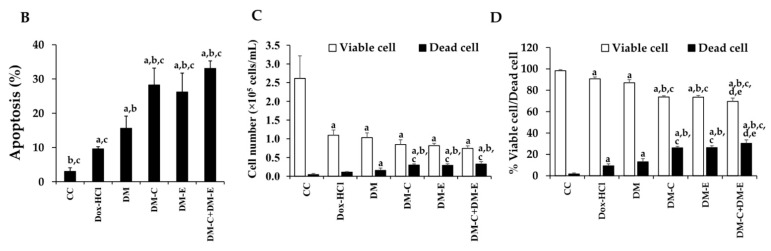
Following 48 h of incubation with media (CC), Dox-HCl, DM, DM-CKR (DM-C), DM-EVQ (DM-E), DM-CKR+DM-EVQ (DM-C+DM-E) at 37 °C, 5% CO_2_. (**A**) The apoptotic rate of KG-1a cells was determined using Annexin V and PI staining and flow cytometry. The quadrants Q1, Q2, Q3, and Q4 indicated necrotic cells, late apoptotic cells, early apoptotic cells, and intact cells, respectively. (**B**) The percentage of apoptotic cells (both early and late apoptosis) in cell control (CC), Dox-HCl, and different DM formulations. (**C**) The cell number and (**D**) the percentage of viable and dead cells of each sample were determined using the trypan blue exclusion method. The data were represented as mean ± SD (*n* = 3). ^a^ *p* < 0.05 compared with CC, ^b^ *p* < 0.05 compared with Dox-HCl, ^c^ *p* < 0.05 compared with DM, ^d^ *p* < 0.05 compared with DM-C, and ^e^ *p* < 0.05 compared with DM-E.

Doxorubicin is known to induce apoptosis in many tumor cells, such as leukemia, breast cancer, and ovarian cancer [21]. In this experiment, to investigate apoptosis induction ability of DM formulations compared with Dox aqueous solution, KG-1a cells were treated with DM with and without peptides and compared with Dox-HCl at a concentration of 0.8 µg/mL (concentration at IC_50_ of Dox-HCl) at 37 °C, 5% CO_2_ for 48 h. Apoptotic cells were detected with flow cytometry using Annexin-VI/PI staining. In comparison with Dox-HCl, the results showed that all DM formulations could considerably stimulate KG-1a cells to enter apoptosis (Figure 12A). Moreover, DM-conjugated with the FLT3 peptides were found to significantly increase apoptosis compared with DM. The apoptosis rates (pro-apoptotic and apoptotic cells) of Dox-HCl solution, DM, DM-CKR, DM-EVQ, and DM-CKR+DM-EVQ treated samples were 9.60 ± 0.62%, 19.14 ± 3.54%, 28.27 ± 4.90%, 26.22 ± 5.51%, and 33.10 ± 2.19%, respectively, when compared with the control (3.04 ± 1.04%) (Figure 12B). Interestingly, the percentage of apoptotic cells in the Dox-HCl treated samples was rather low; thus, the cell number and percentage of viable cells in each sample were evaluated using the trypan blue exclusion method to determine the effect of Dox on cell expansion. The cell number of KG-1a cells decreased from 2.82 ± 0.65 × 10^5^ cells/mL in the control group to 1.14 ± 0.24 × 10^5^, 0.89 ± 0.13 × 10^5^, 0.90 ± 0.17 × 10^5^, 0.82 ± 0.16 × 10^5^, and 1.13 ± 0.13 × 10^5^ cells/mL in response to the treatments of Dox-HCl, DM, DM-CKR, DM-EVQ, and DM-CKR+DM-EVQ, respectively (Figure 12C). The percentage of viable and dead cells was consistent with the presence of apoptotic cells (Figure 12D). Dox-HCl-solution-treated samples had lower cell counts than control samples, with cell viability greater than 90%, showing that at the IC_50_ concentration of Dox-HCl could suppress cell proliferation of AML leukemic stem-cell-like KG-1a cells but not destroy them. As a result, it was proposed that micelle and FLT3 peptides could improve Dox uptake in KG-1a cells, resulting in increased Dox cytotoxic activity.

### 3.10. Cellular Uptake of Dox-HCl and DM Formulations

**Figure 13:**
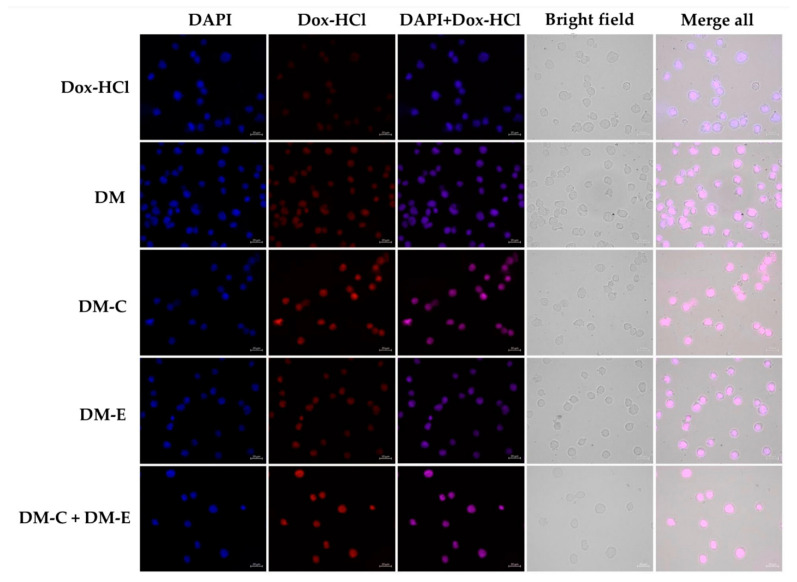
Dox intensity images of Dox-HCl, DM, DM-CKR (DM-C), DM-EVQ (DM-E), and DM-CKR+DM-EVQ (DM-C+DM-E) after 5 h of incubation with KG-1a cells, examined under a fluorescence microscope. Cells were counterstained with DAPI to represent nuclei (scale bar: 20 μm).

To confirm cellular Dox uptake, the cellular uptake of Dox-HCl, DM, DM-CKR, DM-EVQ, and DM-CKR+DM-EVQ were qualitatively observed in KG-1a cells under a fluorescence microscope. In this study, due to the drug resistant property of KG-1a cells, all samples were treated with a high concentration of Dox (3 µg/mL), so we could detect fluorescent signals in the treated cells. After 5 h of incubation time, the cellular fluorescence intensities of Dox in KG-1a cells treated with Dox aqueous solution exhibited weak red fluorescence intensity in the nuclei of cells compared with Dox in the form of micelles, indicating that the use of P407-micelle as a drug carrier could increase drug uptake in KG-1a cells. Additionally, the red fluorescence intensity of Dox in DM conjugated with FLT3 peptides, especially DM-CKR and DM-CKR+DM-EVQ treated cells, was markedly increased compared with that in Dox-HCl, DM, and DM-EVQ groups (Figure 13), which possibly confirmed that the FLT3 peptide could raise cellular uptake of DOX in KG-1a cells. These results were in accordance with the results of MTT and apoptosis.

### 3.11. Effects of Synthesized FLT3 Peptides on FLT3 Protein Expression

**Figure 14:**
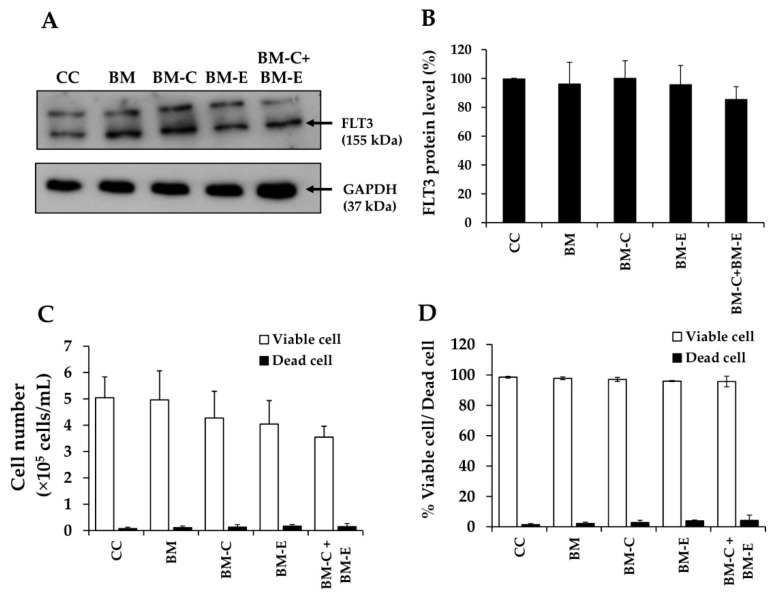
Effects of BM and BM conjugated FLT3 peptides on KG-1a cells. (**A**) FLT3 protein bands, (**B**) percentage of FLT3 protein expression level, (**C**) cell number, and (**D**) cell viability of KG-1a cells after treatment with media (CC, BM, BM-CKR (BM-C), BM-EVQ (BM-E), and BM-CKR+BM-EVQ (BM-C+BM-E)) for 48 h.

FLT3 protein, a member of the class III receptor tyrosine kinase (RTK) family, has been shown to be overexpressed in leukemic cells and to play an important role in their proliferation and survival. Since it was reported that synthesized FLT3 peptides, CKR and EVQ, could bind to the extracellular domains 2 and 3 of the FLT3 receptor, it is necessary to determine whether these peptides affect FLT3 protein expression. The KG-1a cells were treated with BM, BM-CKR, BM-EVQ, and BM-CKR+EVQ at 0.3 mg/mL (the concentration of IC_20_ of BM-CKR+BM-EVQ). After KG-1a cells were incubated with BM and BM attached FLT3 peptides, there was no difference in FLT3 protein expression levels when compared with the control group (Figure 14A). The percentages of FLT3 levels in KG-1a cells treated with CC, BM, BM-CKR, BM-EVQ, and BM-CKR+BM-EVQ were 100 ± 0%, 96.45 ± 14.79%, 100.33 ± 11.90%, 95.97 ± 12.97%, and 85.77 ± 8.43%, respectively (Figure 14B). Furthermore, there was no significant change in total cell number or percentage of viable and dead cells between the control and test groups (Figure 14C,D). Finally, CKR and EVQ peptides had no effect on FLT3 protein expression or cell proliferation.

## 4. Discussion

The optimal conditions were investigated to produce DM micelles to enable intracellular delivery of Dox. Specifically, a film-hydration and pH-induced self-assembly method were explored [39]. Due to its self-assembly in aqueous media, P407 is known to readily form a core-shell micelle structure, whereas Dox has been reported to precipitate in buffers, such as PBS solution, pH 7.4, due to its dimerization [43]. Thus, Dox in aqueous solution was capable of self-assembling into the hydrophobic core structure of the micelles after adding Dox into blank micelles under buffered condition to form Dox-micelles [39,44]. The basic characterization of DMs at several weight ratios of Dox and polymer demonstrated that the weight ratio at 1:200 of DMs showed particle sizes ranging from 22 to 25 nm. The micelles should be small enough (~10–200 nm) to effectively penetrate tissue and avoid clearance by kidney filtration, mononuclear phagocyte system (MPS), or reticuloendothelial system (RES) [45,46]. The surface charges of DMs were close to neutrality (0 to −5 mV); small zeta potential values can cause particle agglomeration and flocculation as a result of van der Waals attractive forces [47]. However, the low zeta potential could be found in several studies on micelles [39,48,49]. The hydrophobic PEO block of P407, which was used as a core-shell micelle, is known to be effective at preventing aggregation, protein absorption, and recognition by RES [32,48]. The percent entrapment efficiency of DMs was greater than 90%, demonstrating that Dox could be encapsulated in the inner core of micelles. This was supported by the amorphous state of Dox and the melting temperature pattern of DM, which matched that of blank micelles. The investigation of drug release also revealed that DM could prolong Dox release time period, which would be advantageous for extending Dox circulation in the body.

Plasma protein adsorption on an intravenous drug carrier is commonly characterized as the factor that determines the in vivo behavior of nanoparticles and microparticles [50]. In this study, DMs were incubated in various concentrations of BSA in PBS, pH 7.4 solution to imitate human plasma condition. The results showed that 90% of average particle size of DMs decreased to 5–20 nm, whereas 10% were found to increase to roughly 150 nm. Although it has been reported that P407 reduces protein adsorption and aggregation [50], the amount of BSA and P407 used in this experiment might have led to the aggregation of some micelles. Since the size of BSA is approximately 7 nm, we were able to detect a large population of small particles. Additionally, P407 had a propensity to aggregate at RT or high temperature. For these reasons, the two populations of particle sizes could be detected after incubating samples in BSA solution at 37 °C for 24 h. Moreover, the effect of temperature on DMs storage demonstrated that freezing micelles at −80 °C could preserve the size and drug content in DM. The long PEO chains of poloxamer act as a cryoprotectant [51]; therefore, the freezing and thawing process had no effect on the physiochemical characteristic of DMs in aqueous solution. Based on these findings, core-shell micelles synthesized from P407 were suitable for transporting Dox.

To improve drug uptake efficacy in AML-LSC, the synthesized FLT3 ligand, CKR and EVQ peptides were conjugated with P407 by CuAAC reaction. In the previous study, curcumin micelle conjugated with CKR and EVQ peptides was reported to enhance solubility and cytotoxicity of curcumin on FLT3 expressing cells [23]. In this study, several DM formulations were prepared using CA-CKR, CA-EVQ, and CA-CKR combined with CA-EVQ to determine the difference in cytotoxicity between DM conjugated with a single or two peptides displayed on the micelle surface. In terms of physiochemical characteristics, there was no difference between size, zeta potential, and Dox content in DM-CKR, DM-EVQ, and DM-CKR+DM-EVQ. Because the molecular weight of CKR (1132.29 g/mol) is similar to that of EVQ (1237.00 g/mol), we also used the same concentration ratio of peptide and CA (1:2) for preparing polymer–peptide conjugation, resulting in the same physiochemical features of these micelle formulations. DM, on the other hand, had a smaller particle size and higher drug content than the conjugation group, indicating that the addition of peptides may have had a small effect on micelle formation [52]. However, the colloid stability data of DM conjugated with peptides was comparable with that of DM without peptides, showing that all DM formulations were feasible to deliver Dox under physiological conditions.

To investigate the effects of DM formulations, AML FLT3-positive cells, leukemic stem cell-like KG-1a cells, and leukemic EoL-1 cells were used to evaluate the cytotoxic effects of Dox solution, DM, and DM-conjugated peptides. DM significantly improved the toxicity of Dox in KG-1a cells compared with Dox-HCl solution. Nanoparticle characteristics, including size, shape, charge, and surface modification, are believed to affect intracellular drug delivery [53]. Previous studies demonstrated that nanomaterials with particle sizes less than 120 nm could be internalized by clathrin-dependent endocytosis, transported to endosomes, and accumulated in lysosomes, whereas the particles in micron scale (0.5–1 µm) were taken up into the cell via macropinosomes and fused with lysosome [54]. Poloxamers, such as P85, whose micelle sizes typically range between 10 and 100 nm, have shown that unimers can be internalized by caveolae-mediated endocytosis, while P85 micelles internalize via clathrin-mediated endocytosis [55]. In addition, PEO blocks in PEO-PPO block copolymers can inhibit drug efflux pumps, P-gp, by altering the microenvironment of the efflux pumps that probably results in fluidity changes in the polar head group regions of cell membranes [56]. Thus, Dox-loaded P407 micelles could improve Dox uptake and extend drug release time in KG-1a cells, which express a variety of ABC transporters, including P-glycoprotein, BCRP, and MRP8, leading to resistance to several chemotherapeutic drugs [57]. Moreover, DM conjugated peptides showed markedly lower IC_50_ values than DM, with DM-CKR+DM-EVQ exhibiting the lowest IC_50_ value in KG-1a cells, indicating that FLT3 peptides might enhance cytotoxicity of DM in leukemic stem cells. KG-1a cells have been shown to be resistant to apoptosis [57]. Corresponding to the results from the cytotoxic assay, DM and DM linked peptides, particularly DM-CKR and DM-CKR+DM-EVQ, were able to improve the apoptotic activity of Dox in KG-1a cells. At the IC_50_ concentration, Dox aqueous solution generated only approximately 10% apoptosis; however, it could promote cell cycle arrest at the G2/M phase, which might have resulted in cell growth inhibition (data not shown). The use of DM and DM conjugated micelles likely enhanced Dox uptake by the cells, resulting in higher toxicity of Dox. Dox accumulation in KG-1a cells was examined by cellular drug uptake. This finding showed that the use of micelles in conjunction with particular peptides could increase Dox uptake and accumulation in targeted cells. Meanwhile, the cytotoxic effects of DM-CKR and DM-CKR+DM-EVQ on KG-1a cells were comparable, although DM-CKR+DM-EVQ was more successful at eradicating KG-1a cells. Tima et al. (2016) demonstrated that CKR-CM-micelles could improve curcumin accumulation in EoL-1 and MV-4-11 cells by a 1.45-fold increase compared with EVQ-CM-micelles [23]. Thus, the CKR peptide might be the major peptide responsible for the peptide–target cell interaction. Nevertheless, the binding location of both CKR and EVQ is on extracellular domains 2 and 3 of the FLT3 receptor [23]. The application of two peptides probably increased the opportunity that micelles had to bind to the FLT3 receptor.

Despite increasing research on polymer micelles as drug delivery vehicles for poorly soluble anticancer drugs, much of the work has been undertaken in a laboratory setting with very few reports in clinical trial studies. The application of polymer micelle drug delivery systems in oral delivery and anticancer therapy is limited to polymeric micelles that encapsulate anticancer drug by physical interactions rather than via chemical linkages. Our study attempted to prepare Dox-loaded micelles with physiochemical stability and then chemically modified these vehicles to conjugate the FLT3-targeting peptides. The use of DM in combination with the FLT3 specific peptides could perhaps improve upon limitations of polymer micelle drug delivery systems.

## 5. Conclusions

Dox-loaded micelles were successfully formulated using Dox aqueous solution and poloxamer 407 at a weight ratio of 1:200 mg. This formulation of DMs exhibited particle sizes in the nanoscale and high Dox encapsulation, with long physiochemical stability at −80 °C. Furthermore, DMs could prolong drug release and maintain their physical properties when incubated in conditions mimicking circulation. FLT3 peptides, CKR and EVQ, were conjugated to a modified P407 or cargo azide to improve DM uptake in FLT3-positive AML cells. DM conjugated peptides also had physiochemical properties similar to DM. For cell assays, DM with and without single and two FLT3 peptides, particularly DM-CKR+DM-EVQ, improved cytotoxicity and induced apoptosis in leukemic stem-cell-like KG-1a cells. The use of P407 micelles in combination with FLT3 peptides could increase Dox uptake, resulting in increased Dox accumulation and the ability to destroy LSCs, which could be a selective approach for developing a new AML-LSC treatment strategy. Thus, the combination of polymeric micelles conjugated with the duo of different peptides may be a new strategic model and new research finding for chemotherapeutic treatment as a drug delivery system in the future.

## Figures and Tables

**Table 1:** Particle size, polydispersity index (PdI), zeta potential (ZP), and %entrapment efficacy (EE) of various formulations of DM on day 0 and particle size on day 4 at RT (*n* = 3, mean ± SE).

Sample	Dox:P407 (mg)	Particle Size (nm)		PdI Day 0	ZP (mV) Day 0	%EE of Dox Day 0	%LC of Dox Day 0
		Day 0	Day 4				
DM1	1:40	25.18 ± 3.24	4440.89 ± 1805.49	0.24 ± 0.01	ND	ND	ND
DM2	1:100	23.44 ± 2.28	451.40 ± 183.65	0.17 ± 0	ND	ND	ND
DM3	1:150	23.98 ± 1.17	259.11 ± 17.24	0.17 ± 0.01	−1.08 ± 1.88	95.76 ± 4.21	0.64 ± 0.03
DM4	1:160	22.16 ± 0.12	129.65 ± 9.64	0.16 ± 0.01	−3.05 ± 4.02	97.81 ± 3.15	0.61 ± 0.02
DM5	1:170	22.43 ± 0.07	180.51 ± 26.35	0.14 ± 0.01	−2.66 ± 2.41	88.98 ± 3.17	0.53 ± 0.02
DM6	1:180	23.03 ± 0.05	176.32 ± 5.18	0.16 ± 0.02	−0.05 ± 4.31	94.13 ± 4.13	0.53 ± 0.02
DM7	1:190	22.55 ± 0.01	119.53 ± 21.82	0.14 ± 0.01	−0.74 ± 3.59	95.75 ± 4.73	0.47 ± 0.03
DM8	1:200	23.33 ± 1.25	54.46 ± 15.98	0.15 ± 0.02	−1.41 ± 2.09	92.85 ± 1.67	0.44 ± 0.01
DM9	1:220	23.21 ± 0.47	57.40 ± 12.67	0.17 ± 0.01	−4.90 ± 7.51	91.76 ± 7.10	0.42 ± 0.03
DM10	1:240	22.04 ± 0.02	39.27 ± 3.52	0.16 ± 0.02	−0.40 ± 0.41	94.40 ± 5.69	0.39 ± 0.02

ND = not done.

**Table 2:** Particle size, polydispersity index (PdI), zeta potential (ZP), % entrapment efficacy (EE)*,* and %loading capacity (LC) of BM-CKR, BM-EVQ, BM-CKR+BM-EVQ, DM-CKR, DM-EVQ, and DM-CKR+DM-EVQ on day 0 at room temperature.

Formulation	Size (mm) Day 0	PdI Day 0	Zp (mV) Day 0	%LC of Dox Day 0	%EE of Dox Day 0
BM-CKR	31.84 ± 0.09	0.19 ± 0.01	−0.23 ± 0.23	ND	ND
BM-EVQ	37.10 ± 0.12	0.28 ± 0.01	−0.52 ± 0.28	ND	ND
BM-CKR+DM-EVQ	30.98 ± 0.03	0.16 ± 0	−1.18 ± 0.98	ND	ND
DM-CKR	38.78 ± 8.11	0.27 ± 0.10	−1.99 ± 0.11	0.36 ± 0.02	71.04 ± 3.80
DM-EVQ	41.25 ± 2.93	0.32 ± 0.04	−2.27 ± 0.04	0.38 ± 0.05	76.92 ± 9.61
DM-CKR+DM-EVQ	34.35 ± 0.97	0.23 ± 0.01	−1.21 ± 0.06	0.36 ± 0.01	71.48 ± 2.47

ND = not done.

**Table 3:** Particle size, polydispersity index (PdI), zeta potential (ZP), and % entrapment efficacy (EE) and %loading capacity (LC) of DM-CKR, DM-EVQ, and DM-CKR+DM-EVQ on day 60 at −80 °C.

Formulation	Size (mm) Day 60	PdI Day 60	Zp (mV) Day 60	%LC of Dox Day 60	%EE of Dox Day 60
DM-CKR	54.03 ± 1.89	0.51 ± 0.03	0.03 ± 0.26	0.28 ± 0.03	55.80 ± 6.68
DM-EVQ	58.12 ± 0.94	0.43 ± 0.01	-0.20 ± 0.87	0.27 ± 0.03	54.82 ± 6.67
DM-CKR+DM-EVQ	53.06 ± 1.05	0.47 ± 0.02	-0.07 ± 0.32	0.31 ± 0.03	61.21 ± 6.84

## Data Availability

Not applicable.
